# Regional association analysis-based fine mapping of three clustered QTL for verticillium wilt resistance in cotton (*G. hirsutum*. L)

**DOI:** 10.1186/s12864-017-4074-y

**Published:** 2017-08-25

**Authors:** Yunlei Zhao, Hongmei Wang, Wei Chen, Pei Zhao, Haiyan Gong, Xiaohui Sang, Yanli Cui

**Affiliations:** State Key Laboratory of Cotton Biology, Institute of Cotton Research of Chinese Academy of Agricultural Sciences (CAAS), Anyang, Henan 455000 China

**Keywords:** Cotton, Verticillium wilt, QTL, Regional association analysis, SNP

## Abstract

**Background:**

Verticillium wilt is one of the most destructive diseases affecting global cotton production. The most effective way to control wilt disease has been the development of new cotton varieties that are resistant to VW. VW-resistant Upland cotton cultivars have been created in both the USA and China by *Gossypium barbadense* introgression. More than 100 VW resistance quantitative trait loci have been detected.

**Results:**

Three clustered VW resistance-related QTL were detected in a 120-line association population and assigned to a genome region of 14,653,469–55,190,112 bp in Dt_chr9. A regional association analysis-based fine-mapping strategy was developed to narrow down the confidence intervals of the above QTL. The estimated LD decay of the genome region of interest was much faster than those of the Dt_chr9 chromosome and the whole genome, suggesting the existence of a recombination hotspot. Thirty-seven haplotype blocks were detected. The confidence intervals of the three clustered QTL were narrowed down to a region of 937,906 bp involving *QTL-i23734Gh* and a region of 1,389,417 bp involving *QTL- i10740Gh*, respectively. Each region contained the strongest association signal. Comparative analysis redefined the confidence intervals of the other three QTLs, *qDL52T2-c19, QTL-BNL4069*, and *QTL-JESPR0001*. The broad-spectrum VW resistance QTL *qVW-D9–1* was demonstrated to be closely linked with the three redefined QTL, *QTL-i23734Gh*, *QTL- i10740Gh* and *QTL-JESPR0001*. Twelve functional genes were detected to be located within the redefined confidence intervals of VW resistance QTL. The mRNA CotAD_60243, encoding E3 ubiquitin-protein ligase UPL2-like, responsible for plant innate immunity and broad-spectrum disease resistance, was found to be overlapped with the strongest association signal i10740Gh. Six mRNAs encoding putative disease-resistance proteins were within the redefined confidence interval of *QTL-JESPR0001*, suggesting a tandem arrangement of R genes.

**Conclusions:**

Our results proved that the VW resistance effect related to three clustered VW resistance-related QTL was actually controled by two redefined major QTL and severlal minor loci. The broad-spectrum VW resistance QTL *qVW-D9–1* may be closely linked with the two redefined major QTLs. The tandem arrangement of R genes were detected in the redefined confidence interval of *QTL-JESPR0001*. The candidate genes obtained should be helpful in identifying and characterizing defense genes related to VW resistance QTL.

**Electronic supplementary material:**

The online version of this article (doi:10.1186/s12864-017-4074-y) contains supplementary material, which is available to authorized users.

## Background

As the most important natural fiber crop in the world, cotton provides approximately 35% of the total fiber used worldwide [1]. There are 46 diploid (2n = 2× = 26) and five allotetraploid (2n = 2× = 52) species in the genus *Gossypium* [2], of which four have been domesticated and cultivated, including two diploids (2n = 2× = 26): *G. arboreum* L. (A_2_A_2_) and *G. herbaceum* L. (A_1_A_1_), and two tetraploids (2n = 4× = 52): *G. hirsutum* (AD_1_AD_1_) and *G. barbadense* (AD_2_AD_2_). Because of their economic importance, *G. hirsutum* and *G. barbadense* are the predominant cultivated species and account for approximately 97% and 3% of cotton production, respectively [3]. Verticillium wilt (VW) caused by the soil-borne fungus *Verticillium dahliae* Kleb. is one of the most destructive diseases in cotton production in the world. VW causes significant decreases in seed-cotton yield and fiber quality [1]. The most effective and feasible way to control wilt disease has been the development of new cotton varieties resistant to VW. Although *G. barbadense* is resistant or tolerant to VW, it has a low yield and is adapted to growth under specific conditions or environments. *G. hirsutum* has high yield and broad environmental suitability but is generally susceptible or only slightly resistant to VW. *G. barbadense* introgression has been used to create VW-resistant Upland cotton cultivars in both the USA and China [1].

The development of molecular quantitative genetics has enabled the direct selection of genotypes by screening molecular markers tightly linked with genes controlling phenotypes of interest. Accordingly, it is important to map the major genes or quantitative trait loci (QTL) for VW resistance in cotton and conduct marker-assisted selection (MAS) for the genetic improvement of disease resistance. More than 100 VW resistance QTL distributed among nearly all 26 tetraploid cotton chromosomes have been detected in different mapping populations [1]. However, the consistency and utility of the above QTL in breeding and genomic research remain uncertain. Because different segregating populations and molecular markers have been used in these studies, the resulting QTL cannot be integrated. Further more, the temporary segregating populations used in these studies, such as F_2_, BC_1_F_1_ and F_2:3_, have led to unrepeatable evaluations of disease resistance for the same genotypes. Fortunately, the availability of tetraploid cotton genomic sequences [4] has facilitated the integration of the results from different QTL mappings and has established a basis for fine mapping of VW resistance QTL of interest.

In addition to QTL mapping, another approach for detecting molecular markers tightly linked with genes controlling phenotypes of interest is association analysis, also known as LD mapping. In contrast to QTL mapping using biparental populations, association analysis is based on linkage disequilibrium (LD) and uses natural populations unrelated by any specific crossing design [5]. Accordingly, association analysis is time- and cost-effective and, more importantly, can be used to investigate the larger number of recombination events occurring in the natural population than in biparental segregating populations, thus achieving a higher resolution of QTL mapping [6]. Association analysis has been widely used in cotton to dissect the genetic basis of complex traits, such as fiber quality traits [7, 8], yield traits [9], salinity tolerance [10] and VW resistance [11].

In this study, we detected three clustered VW resistance-related QTL by both traditional QTL mapping and LD-based association mapping and developed a regional association analysis-based fine-mapping strategy to narrow down the confidence intervals of the above QTL. Our objectives were as follows: (1) to anchor the physical positions of the three clustered VW resistance-related QTL on the cotton AD genome and genotype a cotton panel using SNPs located in the genome region of interest; (2) to estimate the LD and haplotype in the genome region of interest; (3) to conduct fine mapping of VW resistance QTL by SNP-based regional association analysis; and (4) to predict the putative candidate genes related to VW resistance by detecting mRNAs near or overlapping with the peak signal in the confidence intervals of the finely mapped QTL.

## Methods

### Plant materials and trait evaluation

A collection of 329 cotton (*Gossypium hirsutum* L.) accessions from the China cotton germplasm collection were first analyzed and those with same pedigree and similar performance in agronomy traits were excluded, resulting in a panel of 158 cotton accessions reported in our previous study [11]. In this study, the above 158 cotton accessions were further analyzed and those with unambiguous assignment in population structure inference were excluded, resulting in the present association panel consisting of 120 cotton (*Gossypium hirsutum* L.) accessions (Additional file 1: Table S1), which was not genetically highly structured and interrelated yet exhibits high phenotypic diversity, and represented the genetic variation of at least 329 elite *G. hirsutum* cultivar accessions from the China cotton germplasm collection. The 120 cotton lines were planted in both an artificial VW nursery and a greenhouse to evaluate Verticillium wilt resistance at the adult-plant stage and at the seedling stage, respectively. The artificial VW nursery was heavily infected yearly with *Verticillium dahliae* isolate Vd080, a defoliating strain moderately pathogenic to cotton, and the greenhouse had a controlled 12-h photoperiod and temperature variation of 23–30 °C. The experiments were performed in the experimental farm of Cotton Research Institute, Chinese Academy of Agricultural Sciences, Anyang, China in 2009 (for the artificialVW nursery) and 2010 (for the greenhouse), respectively. The experimental designs in both the two environments were randomized blocks with three replications. A susceptible cultivar, Jimian 11 was used as a susceptible control to estimate the severity of disease and determine the optimal time for investigation. Our previous study had showed that the susceptible cultivar Jimian 11 had heavily and uniform symptoms of verticillium wilt upon the infection of *Verticillium dahliae* in both the artificial VW nursery and the greenhouse, regardless of years in which the experiments were performed. The infection experiments in the two environments were performed according to the description in our previous study [11]. VW resistance was evaluated by using the relative disease index (RDI), which is an adjustment of the disease index to decrease the error of investigation in different environments by contrasting the disease index of tested samples with the disease index of the susceptible control mentioned above [11]. Analysis of variance and descriptive statistics were performed using the SAS system (version8.02, SAS Institute Inc., Cary, NC, USA). A correlation analysis was performed to evaluate the correlation between the trait performances in the two environments.

### Detection of major QTL and anchoring of physical positions

Both sequences of the end markers of the three clustered VW resistance-related QTL, *qV-VD8M-D9–1*, *qV-BP2S1-D9–1* and *qVL-D5-1BC*_*1*_ *S*_*2*_ *592*, detected using traditional QTL mapping by Jiang et al. (2009) and Yang et al. (2008) [12, 13], were assigned to the cotton AD genome in the TM-1 (*Gossypium hirsutum* L.) genome sequencing project (Sequencing version: BGI_Gossypium_hirsutum_v1.0, https://www.cottongen.org/species/Gossypium_hirsutum/bgi-AD1_genome_v1.0), thus resulting in an uninterrupted genome region of 14,653,469–55,190,112 bp in Dt_chr9 (Additional file 2: Table S2). All sequences of SSR markers associated with VW resistance detected using association mapping in our previous study [11] were also assigned to the same genome (Additional file 2: Table S2), and we obtained three VW resistance-related markers, NAU980, NAU5064 and JESPR0001, located in the genome region of 14,653,469–55,190,112 bp in Dt_chr9, thus demonstrating the existence of three clustered QTL in this 120-line association population.

### SNP genotyping and in silico mapping

The 120 cotton lines were genotyped by using Illumina Cotton70kBeadChips (Illumina, USA) according to the manufacturer’s protocol, using a total of 50 ng of genomic DNA. Raw hybridization intensity data processing and genotype calling were performed usingthe software GenomeStudio (v2011.1, Illumina®). SNPs with a minor allelic frequency (MAF) >0.05 and call rate > 0.9 were retained and used in further analysis, thus resulting in a final total of 21,171 SNPs. The 21,171 source sequences in which the 21,171 SNPs were identified were mapped in silico onto the cotton genome through a BlastN search against the cotton AD genome from the TM-1(*Gossypium hirsutum* L.) genome sequencing project (Sequencing version: BGI_Gossypium_hirsutum_v1.0, https://www.cottongen.org/species/Gossypium_hirsutum/bgi-AD1_genome_v1.0). Only the top blast hits against the source sequences were considered, on the basis of an e-value threshold of e^−18^. The SNPs located in the genome region of 14,653,469–55,190,112 bp in Dt_chr9 were screened, and their corresponding genotypes were obtained by extracting the SNP genotypes from the chip-hybridized genotypes of the 120 lines.

### Linkage disequilibrium and regional association analysis

LD was estimated by calculating *r* ^*2*^ (average correlation coefficient) between all pairs of SNP markers among 120 cotton accessions by using the software package TASSEL3.0 [14]. The association between LD decay and physical distances in the genome region of interest in the *Gossypium hirsutum* L. genome was evaluated by fitting a nonlinear model according to the description by Li et al. (2014) [15]. The haplotype blocks were estimated and visualized using Haploview software version 3.32 [16]. The structure of the 120 cotton accessions was inferred using the software STRUCTURE v 2.3.4 [17]. The K-value (the putative number of clusters) was set from 1 to 10, and the iteration number was set to 3. A burn-in period of 10,000 followed by 100,000 replications of Markov Chain Monte Carlo was used when running STRUCTURE. The optimal number of clusters was determined on the basis of the posterior probability [Ln P(D)] of each K and an ad hocmeasure *Δk* based on the relative rate of change in Ln P(D) between successive k [18]. The pairwise kinship estimates were calculated using TASSEL v3.0 software. Regional association analysis was conducted using SNP markers within the genome region of interest in the association population of 120 cotton accessions. We used a mixed linear model (MLM) [19] to incorporate information about population structure (Q) and familial relationship (K) [20] and estimate the association between SNPs and disease-resistance traits. SNPs with *P* values below the significance threshold were compared with the genome region of interest to evaluate candidate disease-resistance genes located in the three clustered QTL regions.

## Results

### Phenotypic analysis of verticillium wilt resistance

**Fig. 1:**
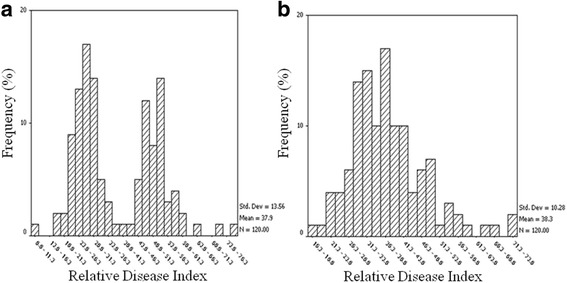
Histogram of the relative disease index of the 120 cotton lines identified in the field disease nursery (**a**) and in the greenhouse (**b**)

The cotton lines, including 82 lines from China, 30 lines from the USA, 3 lines from former Soviet Union, 2 lines from Africa, 1 line from France, 1 line from Australia and 1 line from Pakistan (Additional file 1: Table S1), were phenotyped for their VW resistance in the disease nursery and in the greenhouse. The relative disease index (RDI) was obtained for the 120 cotton lines. The histogram of RDI for the 120 cotton lines revealed a wide range of phenotypic variation of VW resistance both in the disease nursery and in the greenhouse (Fig. 1). The results of an ANOVA showed that there were significant differences (*P* < 0.01) in VW resistance among the 120 lines (Table 1). The mean trait performances between the two environments were not significant in ANOVA (Table 1), implying a weak environment effect, and there were small significant correlations (*r* = 0.311, *p* < 0.01) for the VW resistance between the two environments.

**Table 1:** ANOVA of the relative disease indexes of the 120 cotton lines in the field disease nursery and in the greenhouse environment

Source	DF	Mean Square	F Value	Pr > F
Genotype	119	184.5815	1.7601^**^	0.0011
Environment	1	11.4954	0.1096	0.7412
Error	119	104.871		

**: significant at *P* < 0.01

### Genotype analysis of the 120 cotton lines on the basis of SNPs located in the genome region of interest

A total of 63,058 SNPs were included in the Cotton70kBeadChip, of which 21,171 SNPs (34%) were successfully called in the 120 lines with less than10% missing data and an MAF of greater than 0.05. In a BlastN search against the cotton AD genome, 18,726 SNPs had top blast hits with an e-value threshold of e-18, with good coverage across the 26 chromosomes, ranging from 1410 SNPs on At_chr7 to 4484 SNPs on Dt_chr1 (Table 2). Screening the physical position information of the SNPs revealed that 2252 SNPs were located in the genome region of 14,653,469–55,190,112 bp in Dt_chr9 (Additional file 3: Table S3), and the corresponding SNP genotypes were obtained from the chip-hybridized genotypes of the 120 lines, thus resulting in a 2252 × 120 matrix consisting of SNP genotypes of 120 cotton lines (Additional file 3: Table S3).

**Table 2:** Number of informative SNPs located on the 26 chromosomes of the cotton AD genome

Chr.^a^	SNPnumber^b^	UniqueSNPs^c^
At_chr1	3506	326
At_chr2	2888	249
At_chr3	3163	144
At_chr4	3757	473
At_chr5	3439	229
At_chr6	2834	186
At_chr7	1410	77
At_chr8	3066	185
At_chr9	3597	322
At_chr10	3267	215
At_chr11	3507	229
At_chr12	2897	135
At_chr13	3492	300
Dt_chr1	4484	683
Dt_chr2	2651	318
Dt_chr3	2112	185
Dt_chr4	2350	238
Dt_chr5	2987	516
Dt_chr6	2379	244
Dt_chr7	2321	111
Dt_chr8	2628	363
Dt_chr9	3435	404
Dt_chr10	2623	265
Dt_chr11	2748	222
Dt_chr12	1987	126
Dt_chr13	2868	300

^a^The chromosome names were based on the TM-1(*Gossypium hirsutum* L.) genome sequencing version BGI_Gossypium_hirsutum_v1.0: https://www.cottongen.org/species/Gossypium_hirsutum/bgi-AD1_genome_v1.0

^b^The informative SNPs were numbered on the basis of the SNPS with an e-value threshold of less than e-18 in a BlastN search against the cotton AD genome

^c^Unique SNPs were regarded as those with only one hit against the genome in Blast

### Estimation of LD and haplotype in the genome region of interest

**Fig. 2:**
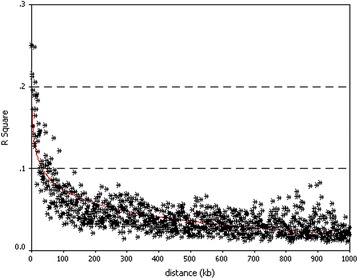
Linkage disequilibrium (LD) decay plot of the genome region of interest of Dt_chr9in cotton. The LD,measured as R squared, between pairs of SNPs is plotted against the distance between the SNPs. For the genome region of interest, LD decayed within 33 kb

On the basis of the 2252 SNPs located in the genome region of interest, the LD structure in the genome region of 14,653,469–55,190,112 bp in Dt_chr9 was investigated by using the genotype data of the 120 lines. The square of the correlation coefficient (*r* ^*2*^) between all pairs of SNPs was calculated using TASSEL software (Additional file 4: Table S4). Triangle plots of pairwise LD between SNP markers demonstrated significant LD blocks in LD analysis of the genome region of interest (Additional file 5: Figure S1). The sizes of these LD blocks or the so-called LD delay in the genome region of interest were identified by plotting *r* ^*2*^, calculated between each pair of SNP loci, against the distance in kilobases between these loci using nonlinear regression. Figure 2 shows the LD delay for this cotton panel in the genome region of interest, in which a significant LD between pairs of SNPs within a distance of 33 kb (*r* ^*2*^ = 0.1) was observed. An analysis using Haploview software partitioned the genome region of interest into 37 haplotype blocks (Additional file 6: Figure S2). The tagged SNPs in the haplotype blocks were those tightly linked on the basis of physical distance. The largest haplotype block contained 7 SNPs, the smallest block contained only 2 SNPs, and the average SNP number in a block was 2.91. The size of the haplotype blocks ranged from 1 kb to 99 kb (Table 3).

**Table 3:** Sizes, SNP numbers and physical positions of haplotype blocks in the genome region of interest

Haplotype block	No.of SNPs	Size of haplotype blocks (kb)	Tagged SNPs	Physical regions of haplotype blocks (bp)
1	2	3	i41355Gh i31583Gh	14,957,075–14,960,858
2	2	5	i21245Gh i24363Gh	15,861,607–15,866,716
4	2	1	i23752Gh i40299Gh	16,224,058–16,225,318
5	2	1	i20303Gh i20304Gh	18,847,855–18,849,240
6	2	18	i38154Gh i32970Gh	27,768,314–27,786,496
7	2	2	i27379Gh i42605Gh	28,871,439–28,873,835
9	2	12	i18314Gh i34016Gh	30,315,506–30,327,684
10	2	3	i07851Gh i16246Gh	31,607,896–31,611,204
11	5	66	i10039Gh i21752Gh i16837Gh i10042Gh i10043Gh	31,719,976–31,786,217
12	2	3	i12267Gh i12269Gh	33,229,157–33,232,678
14	5	82	i00119Gh i00140Gh i00141Gh i16817Gh i43183Gh	34,501,252–34,583,840
15	2	5	i20134Gh i20135Gh	36,073,111–36,078,743
16	3	22	i17680Gh i37718Gh i12365Gh	36,232,823–36,255,525
17	2	7	i43773Gh i17688Gh	36,696,056–36,703,949
19	2	9	i43694Gh i49350Gh	40,180,546–40,190,360
20	2	2	i15894Gh i06726Gh	40,908,038–40,910,540
21	2	1	i08629Gh i08630Gh	41,311,879–41,313,033
22	6	18	i06677Gh i06676Gh i15881Gh i06672Gh i06671Gh i06669Gh	41,859,643–41,878,504
23	3	1	i15880Gh i15879Gh i06667Gh	41,990,968–41,992,942
24	2	6	i14080Gh i19256Gh	42,064,655–42,071,032
25	2	2	i12647Gh i17790Gh	42,778,237–42,778,372
26	6	86	i12646Gh i20168Gh i41612Gh i28975Gh i17786Gh i17783Gh	42,778,734–42,865,091
27	3	2	i02170Gh i02171Gh i18366Gh	44,362,364–44,364,438
28	2	9	i34876Gh i28109Gh	46,465,757–46,475,660
29	2	4	i35043Gh i19582Gh	46,957,845–46,962,126
30	3	3	i00205Gh i09683Gh i16742Gh	48,055,522–48,059,062
31	2	3	i09656Gh i41953Gh	48,745,715–48,749,280
32	2	2	i09626Gh i19574Gh	49,309,585–49,312,204
33	4	53	i02953Gh i02943Gh i14755Gh i02942Gh	51,133,864–51,187,532
34	7	99	i09580Gh i16721Gh i09571Gh i46804Gh i38553Gh i09567Gh i09564Gh	51,907,040–52,006,561
35	4	16	i14164Gh i14163Gh i09551Gh i47386Gh	52,246,626–52,262,912
36	4	86	i09541Gh i22890Gh i00260Gh i19566Gh	52,646,775–52,732,926
37	2	10	i26632Gh i16725Gh	53,386,560–53,397,023

### Regional association mapping

**Fig. 3:**
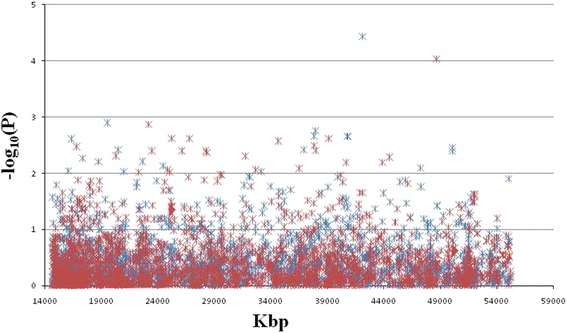
Manhattan plots showing the regional association mapping ofverticillium wilt resistance using 2253 SNPs from the target genome regionon Dt_chr9in cotton(*Gossypium hirsutum* L.). The *blue asterisks* depict the results of the field disease nursery environment, and the *red asterisks* depict the results of the greenhouse environment

The population structure (Q matrix) was determined according to the change in both the posterior probability [LnP(D)] of each K and an ad hoc measure*Δk* (Additional file 7: Figure S3). Considering the population structure (Additional file 8: Table S5) and family relatedness (Additional file 9: Table S6) within the population, regional association analysis was conducted with a mixed linear model (MLM) using 2252 SNPs from the target genome region. In total, 192 statistically significant SNPs were identified to be associated with VW resistance (*P* < 0.05), including 83 identified in the disease nursery environment and 109 in the green house environment (Additional file 10: Table S7). Among them, 10 association signals for VW resistance were shared in both environments (Additional file 10: Table S7), and two showed the strongest association signals in the disease nursery environment (*P* = 3.6E-05 and R^2^ = 15.9%) and in the green house environment (*P* = 9.3E-05 and R^2^ = 17.9%) (Additional file 10: Table S7 and Fig. 3). The strongest association signal (i23734Gh) in the disease nursery environment was located within the two above mentioned QTL, *qV-VD8M-D9–1* and *qVL-D5-1BC*_*1*_ *S*_*2*_ *592*, and was very near NAU980 and NAU5064, the two VW resistance-related markers (Additional file 2: Table S2 and Additional file 10: Table S7). The strongest association signal (i10740Gh) in the greenhouse environment was located within *qV-BP2S1-D9–1* and *qV-VD8 M-D9–1* (Additional file 2: Table S2 and Additional file 10: Table S7).

**Fig. 4:**
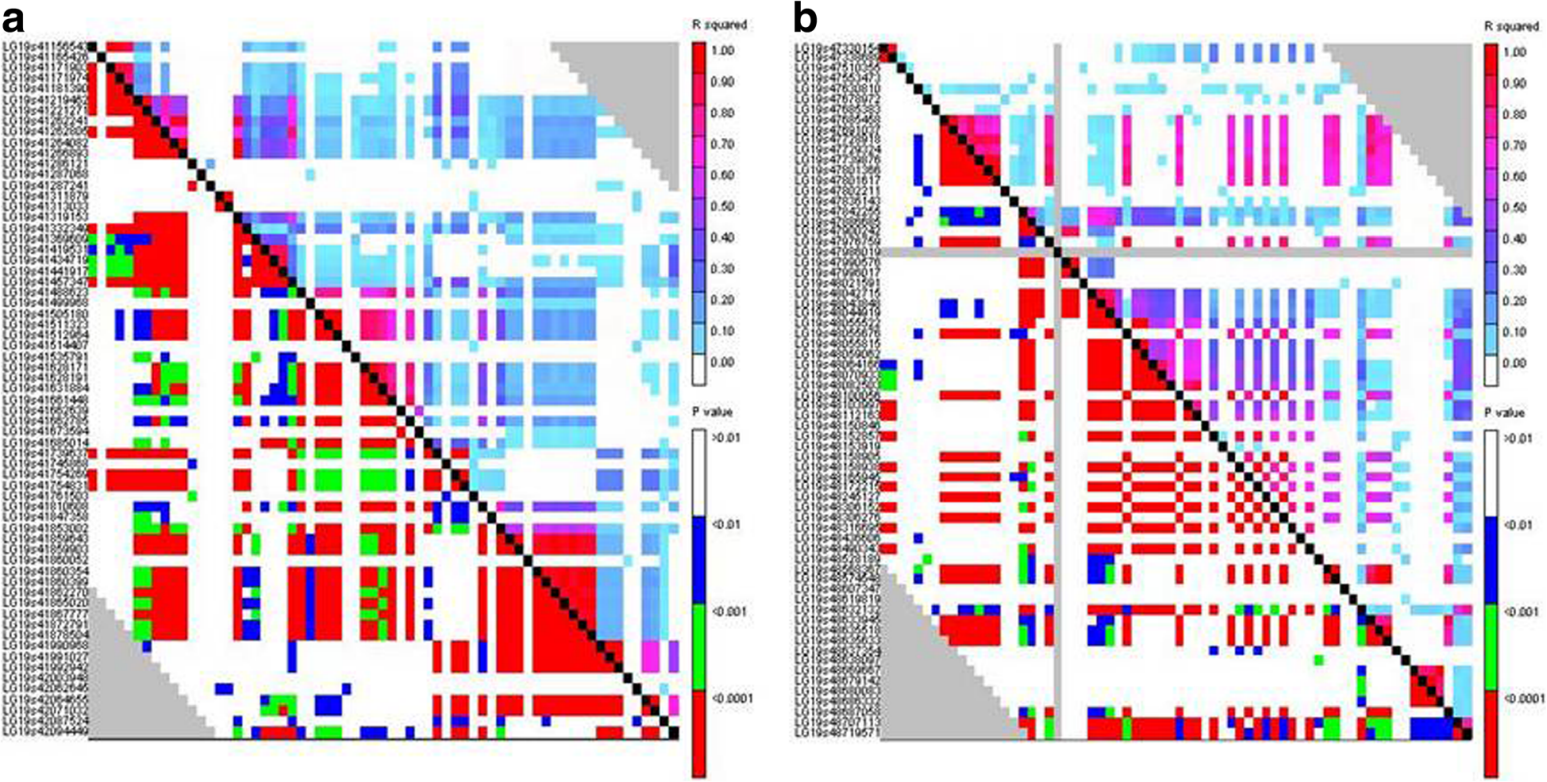
The LD blocks around the strongest associated signals i23734Gh (**a**) and i10740Gh (**b**), which span the distance from 41,156,543 bp to 42,094,449 bp and from 47,330,154 bp to 48,719,571 bp, respectively

To determine the potential QTL regions near the strongest association signals, the extent of the LD around the strongest associated signals (i23734Gh and i10740Gh) was investigated. For convenience, we named the QTL around the strongest association signals i23734Gh and i10740Gh as *QTL- i23734Gh* and *QTL- i10740Gh*, respectively. Eight and three SNPs showed significant LD with i23734Gh (at 42,087,524 bp) and i10740Gh (at 48,637,354 bp) (*P* < 0.01), respectively (Additional file 4: Table S4). The eight SNPs that showed significant LD with i23734Gh were located in several LD blocks between 41,156,543 and 42,094,449 (Additional file 4: Table S4 and Fig. 4a), among which 5 SNPs (i06680Gh at 41,754,269 bp, i06679Gh at 41,754,831 bp, i00366Gh at 41,853,002 bp, i23734Gh at 42,087,524 bp, and i06664Gh at 42,096,123 bp) showed significant association signals with VW resistance (Additional file 10: Table S7). We therefore defined *QTL- i23734Gh* as the region between the SNPs i06714Gh (at 41,156,543 bp) and i06665Gh (at 42,094,449 bp). We further detected 4 haplotype blocks (haplotype block 21, 22, 23 and 24) within the region of this defined QTL (Table 3). The strongest association signal i10740Gh (at 48,637,354 bp) showed significant LD with 3 SNPs (Additional file 4: Table S4), which were located in several LD blocks between 47,330,154 and 48,719,571 (Additional file 4: Table S4 and Fig. 4b). The three SNPs (i40266Gh at 48,669,667 bp, i00554Gh at 48,686,332 bp, i10740Gh at 48,637,354 bp) showed significant association signals with VW resistance, and therefore the potential region of *QTL- i10740Gh* was regarded as the region between the SNP i00548Gh (at 47,330,154 bp) and i09655Gh (at 48,719,571 bp). One haplotype block (haplotype block 30) was located in this region (Table 3).

In addition to the five haplotype blocks located in the regions of the above defined QTL, we detected another 3 haplotype blocks containing significant association SNPs, including haplotype block 11 containing i21752Gh, haplotype block 33 containing i02943Gh, and haplotype block 34 containing i09580Gh, i16721Gh, i16720Gh, i32389Gh, i09578Gh, i09577Gh, i09575Gh, i09574Gh, i09573Gh, i09572Gh, i09571Gh, i26387Gh, i46804Gh, i21925Gh, i09569Gh, i09567Gh, i09565Gh, i16717Gh and i09564Gh (Table 3 and Additional file 10: Table S7). We further compared the 192 statistically significant SNPs with the genome region of interest to detect candidate genes associated with VW resistance. Thirty-six SNPs (22 identified in the disease nursery environment and 18 in the greenhouse environment) were located within the genome regions of 45 functional genes (Additional file 11: Table S8). The homologies of these genes involved disease-resistance proteins (7), protein kinases (4), transcription factors (2), and enzymes involved in biological pathways including protein metabolism, transport complexes and stress reactions (Additional file 11: Table S8).

### Comparative analysis of VW-resistance QTL in the genome region of interest

To compare the VW-resistance loci identified in the present findings with the results of a meta-analysis of VW-resistance QTL identified in previous studies [3], all VW-resistance QTL in chromosome 19 of the consensus map (http://www2.cottonqtldb.org:8081/search) were aligned to the cotton AD genome (Sequencing version: BGI_Gossypium_hirsutum_v1.0,https://www.cottongen.org/species/Gossypium_hirsutum/bgi-AD1_genome_v1.0) by using the sequences of markers in the confidence interval (Table 4). Of the nine QTL in chromosome 19, four were successfully located in the genome region of interest (Table 4), among which *qVL-D5-1BC*_*1*_ *S*_*2*_ *592* was the same as mentioned above. The confidence intervals of the other three QTL, *qDL52T2-c19*, *QTL-BNL4069*, and *QTL-JESPR0001*, were redefined according to the physical positions of the nearest SNPs showing significant association signals with VW resistance (*P* < 0.01). For the QTL *qDL52T2-c19*, the nearest SNP showing significant association signals with VW resistance (*P* < 0.01) was i12373Gh (at 36,482,160 bp), but i12373Gh showed significant LD with i17680Gh (at 36,232,823 bp), i17681Gh (at 36,232,929 bp), i12362Gh (at 36,233,062 bp), i37718Gh (at 36,236,671 bp), i12363Gh (at 36,244,807 bp), i12364Gh (at 36,254,883 bp), i12365Gh (at 36,255,525 bp), i17682Gh (at 36,257,003 bp), i17683Gh (at 36,257,095 bp), and i28415Gh (at 36,302,807 bp). We therefore redefined the confidence interval of this QTL as the region from i17680Gh (at 36,232,823 bp) to i12373Gh (at 36,482,160 bp) (Table 4 and Additional file 10: Table S7). For *QTL-BNL4069*, the nearest SNP showing significant association signals with VW resistance (*P* < 0.01) was i33882Gh (at 26,114,594 bp), but i33882Gh showed significant LD with i40057Gh (at 25,242,321 bp). We therefore redefined the confidence interval of this QTL as the region from i40057Gh (at 25,242,321 bp) to i33882Gh (at 26,114,594 bp) (Table 4 and Additional file 10: Table S7). For *QTL-JESPR0001*, the nearest SNPs showing significant association signals with VW resistance (*P* < 0.01) were i21752Gh (at 31,729,283 bp) and i44041Gh (at 32,632,521 bp). Accordingly, we redefined the confidence interval of this QTL as the region from i21752Gh (at 31,729,283 bp) to i44041Gh (at 32,632,521 bp) (Table 4 and Additional file 10: Table S7).

**Table 4:** Comparative analysis of VW-resistance QTLs in the genome region of interest

Verticillium wilt resistance-related QTLs	Confidence interval or nearest markers	Position in *G. raimondii* genome			Position in *G. hirsutum* genome^a^		
		Chr.of G.r.	start (bp)	end (bp)	Chr.of G.h.	start (bp)	end (bp)
*qVV-D5-1BC*_*1*_ *S*_*2*_ *592*	BNL2656–BNL1671	-	-	-	-	-	-
*qDR85T1-c19*	SHIN-0827-DPL1938	Chr09	70,170,721	70,174,674	-	-	-
*qDL52T2-c19*	HAU006-SNP0208	Chr09	33,835,949	46,015,278	Dt_chr9	31,717,527	35,815,114
*qDR15T1-c19*	SNP0315-SNP0159	Chr09	4,280,669	4,766,613	Dt_chr9	66,815,821	67,323,519
*qVL-D5-1BC*_*1*_ *S*_*2*_ *592*	NAU2513–BNL1878	Chr09			Dt_chr9	14,653,469	46,609,148
*qVWI12-c19.1*	RLKR7–380–RLK-G-520	-	-	-	-	-	-
*QTL-BNL4069*	BNL4069	Chr09			Dt_chr9	26,272,316	26,271,919
*qVV-D5-1BC*_*1*_ *S*_*2*_ *VD8*	NAU1042–NAU828b	Chr09			Dt_chr9	55,189,800	-
*QTL-JESPR0001*	JESPR0001	Chr09			Dt_chr9	32,274,282	-

^a^Positions in the *G. hirsutum* genome were inferred by aligning the sequences of markers in the confidence interval to the cotton AD genome(Sequencing version: BGI_Gossypium_hirsutum_v1.0,https://www.cottongen.org/species/Gossypium_hirsutum/bgi-AD1_genome_v1.0). The sequences of the markers in the confidence interval were obtained based on the physical positions of the SNPs in the *G. raimondii* genome, as described by Gore et al. (2013) or on the basis of the sequences of SSRs searched in the database (https://www.cottongen.org)

## Discussion

Up to now, more than 100 VW resistance QTL have been detected [3, 11–13, 21–28], distributed on almost all 26 tetraploid cotton chromosomes [1]. In this study, by assigning the above QTL to the cotton AD genome, we found a genome region of interest of 14,653,469–55,190,112 bp in Dt_chr9, which contains both three clustered QTL for VW resistance from traditional QTL mapping and three VW resistance-related markers from association mapping (Additional file 2: Table S2). Interestingly, comparative analysis of the VW resistance QTL in the above genome region of interest further demonstrated the existence of another three VW resistance QTL in this region (Table 4). Therefore, a total of 6 QTL were identified in the genome region of interest. Of the 6 QTL, *qV-VD8M-D9–1* and *qV-BP2S1-D9–1* were detected on c23 (D9) by Jiang et al. (2009) and explained 13.1%–24.5% of the phenotypic variance [12], *qVL-D5-1BC*_*1*_ *S*_*2*_ *592* was detected on D5 (chr.19) by Yang et al. (2008) and explained 14.1% of the phenotypic variation [13], *qDL52T2-c19* was detected on chromosome c19 by Fang et al. (2014) and explaining nearly 25% of phenotypic variation [29], *QTL-BNL4069* and *QTL-JESPR0001* were detected on chr.19 by Zhao et al. (2014) and showed significant association with VW resistance [11]. The high contributions of the above QTL implied that the genome region of interest could exist major QTL which could be detected repeatly in different environmental conditions. The different QTL were identified for the same genome region of interest might be due to the low genome coverage of molecular markers in previous mapping studies. In this study, the genome coverage of molecular markers were greatly improved by SNP-based regional association analysis, resulting in a high-resolution detection of QTL for the genome region of interest.

The estimated LD decay of the current *G. hirsutum* association panel in the genome region of interest was 33 kb, which corresponds to approximately 0.08 cM with 400 kb per cM [30], values much lower than those estimated (1–25 cM) in previous studies of cotton [7–9]. Moreover, the LD decay was much faster for the genome region of interest (33 kb) than for the Dt_chr9 chromosome (590 kb) and the whole genome (7200 kb) (our unpublished data), thus suggesting that the genome region of interest should lie within a recombination hotspot. In fact, according to Table 4, the genome region of interest overlapped with a resistance QTL hotspot for VW on chromosome c19 identified by a meta-analysis of QTL [3]. This result also indicated that the QTL within the genome region of interest could be fine mapped through LD mapping with the current association population.

It has been reported that haplotype analysis might provide more detection power than single-marker GWAS and may be more practical for breeding [31]. The LD-based haplotype build method partitioned the genome region of interest into 37 haplotype blocks involving 95 tagged SNPs (Table 3). A total of 8 haplotypes were found to be related to VW resistance, of which five were located in the fine-mapped QTL regions and three contained significant association signals with VW resistance, including the 99-kb-long haplotype block 34, which contains19 SNPs significantly associated with VW resistance. This result suggested that haplotype-based GWAS has a higher marker detection efficiency if furnished sufficient marker density.

Approximately 193 QTL for VW resistance have been located in cotton using biparental mapping populations [3], but fine mapping and narrowing down reports have been limited. In this study, we proposed an SNP-based regional association mapping strategy to directly fine map interested QTL. With this strategy, the confidence intervals (from 14,653,469 bp to 55,190,112 bp, approximately 101.34 cM) of three clustered QTL for VW resistance on Dt_chr9 were further narrowed down to a region of 937,906 bp (approximately 2.34 cM, from 41,156,543 bp to 42,094,449 bp on Dt_chr9) and a region of 1,389,417 bp (approximately 3.47 cM, from 47,330,154 bp to 48,719,571 bp on Dt_chr9). We detected at least 5 and 3 significant association signals with VW resistance in the two regions, respectively. Furthermore, we redefined the confidence intervals of the other three QTL aligned onto the genome region of interest in Dt_chr9 according to the physical positions of the nearest SNPs showing significant association signals with VW resistance and the extent of the LD around the nearest SNPs. We redefined the confidence interval of *qDL52T2-c19* as the region from 36,232,823 bp to 36,482,160 bp (approximately 0.62 cM), *QTL-BNL4069* as the region from 25,242,321 bp to 26,114,594 bp (approximately 2.18 cM), and *QTL-JESPR0001* as the region from 31,729,283 bp to 32,632,521 bp (approximately 2.26 cM) (Table 4 and Additional file 10: Table S7). Thus, we redefined a total of six VW resistance QTL in the genome region of interest.

Zhang et al. [3, 32] reported one QTL hotspot for resistance to VW on chromosome c19 (at 0–25 cM on the consensus map), which carried 7 VW resistance QTL. Of the seven QTL, four were successfully located in the genome region of interest (Table 4). By aligning to the cotton AD genome, we proved another two QTL, *qV-VD8M-D9–1* and *qV-BP2S1-D9–1* also located in the genome region of interest (Additional file 2: Table S2). Therefore, we infered that the QTL hotspot on chromosome c19 at least carried 9 VW resistance QTL, among which 6 QTL were located in the genome region of interest. In this study, by regional association analysis-based fine mapping, we further narrowed down the confidence intervals of the above six QTL, and obtained two major QTL showing the strongest association signals and three minor loci showing significant association signals with VW resistance. It should be pointed out that the three QTL, *qV-VD8M-D9–1*, *qV-BP2S1-D9–1* and *qVL-D5-1BC*_*1*_ *S*_*2*_ *592* were not mapped on the same genome regions by Jiang et al. and Yang et al. [12, 13], because of different segregating populations used and low genome coverage of molecular markers. However the current study mapped them in the QTL hotspot on chromosome c19 and redefined them as two major QTL with greatly decreased confidence interval, demonstrating the fact that SNP-based regional association analysis can realize the integration and fine mapping of VW resistance QTL. Besides the two major QTL, another three redefined QTL only showed a weak significant association signals with VW resistance, which implying that the VW resistance effect related to the genome region of interest was actually controled by two major QTL and several minor loci.

Ning et al. [22] reported a major broad-spectrum VW resistance QTL (*qVW-D9–1*) located on chr.D9, which explained 62.83% of the phenotypic variation on the average. In order to figure out whether this QTL was closely linked with the genome region of interest, we assigned this QTL to the cotton AD genome (Sequencing version: BGI_Gossypium_hirsutum_v1.0), and found that one end marker (NAU2954) of this QTL was mapped to the physical position of 58,952,814–58,952,858 bp on Dt_chr9, just outside of the genome region of interest. Considering two TM-1 sequencing versions was published [4, 32], we further assigned both this QTL and the five redefined QTL in the genome region of interest to another TM-1 genome sequenc (Sequencing version: NBI_Gossypium_hirsutum_v1.1, https://www.cottongen.org/species/Gossypium_hirsutum/nbi-AD1_genome_v1.1), and found that the end marker (NAU2954) of QTL *qVW-D9–1* was located in the regions of 42,608,280–42,609,097 bp on chromosome D09, while the five redefined QTL were located in the regions of 42,313,370–42,323,548 bp (for *QTL-i23734Gh*), 42,314,300–42,322,229 bp (for *QTL- i10740Gh*), 42,313,733–42,323,549 bp (for *QTL-JESPR0001*), 28,817,011–28,819,375 bp (for *qDL52T2-c19*) and 10,917,337–10,911,809 bp (for *QTL-BNL4069*) on chromosome D09, respectively. It is evident that the broad-spectrum VW-resistant QTL *qVW-D9–1* was closely linked with *QTL-i23734Gh*, *QTL- i10740Gh* and *QTL-JESPR0001*, and the physical distance between them was about 300 kb.

By comparing the 192 statistically significant SNPs with the genome region of interest, we detected a total of 36 SNPs located within 45 functional genes (Additional file 11: Table S8), including 12 functional genes located within the redefined confidence intervals of VW-resistance QTLs. The mRNAs CotAD_02752 (containing SNP i00366Gh) and CotAD_02779 (containing SNP i06664Gh) were located within the confidence interval of *QTL- i23734Gh*, and approximately 234.5 kb and 8.6 kb from the peak SNP of i23734Gh (Additional file 10: Table S7 and Additional file 11: Table S8). The mRNAs CotAD_60243 (containing SNP i10740Gh), CotAD_60247 (containing SNPi00554Gh) and CotAD_60242 (containing SNPi00538Gh) were located within the confidence interval of *QTL- i10740Gh* (Additional file 11: Table S8). Interestingly, the mRNA CotAD_60243 overlapped with the peak SNP of i10740Gh and encodes E3 ubiquitin-protein ligase UPL2-like, which had been reported to be responsible for plant innate immunity and broad-spectrum disease resistance [33]. The mRNACotAD_08074 (containing SNP i12373Gh) was located within the redefined confidence interval of *qDL52T2-c19* and encodes ROOT PRIMORDIUM DEFECTIVE 1-like protein, a novel plant-specific family gene required for the maintenance of active cell proliferation [34]. The mRNAs CotAD_22101 (containing SNP i16842Gh, i16844Gh, and i26445Gh), CotAD_22100 (containing SNPsi16842Gh, i16844Gh, and i26445Gh), CotAD_22103 (containing SNPsi16842Gh, i16844Gh, and i26445Gh), CotAD_22107 (containing SNPsi16842Gh and i16844Gh), CotAD_22075 (containing SNP i16842Gh), and CotAD_22106 (containing SNP i26445Gh), all of which encode putative disease-resistance proteins (Additional file 11: Table S8), were located within the redefined confidence interval of *QTL-JESPR0001*, thus suggesting a tandem arrangement of R genes.

The identification of candidate genes is a very complicated process. The molecular functions of the candidate genes must be verified by more comprehensive analyses in future studies. Combining the results of the current regional association analysis with the results of previous transcriptome profiling studies may be a promising approach to quickly identify candidate genes [35–38].

## Conclusion

Our results proved the feasibility of regional association analysis-based fine mapping and achieved fine mapping and gene detection for three clustered VW resistance-related QTL. We also redefined the confidence intervals of three other QTL according to the physical positions of the nearest SNPs showing significant association signals with VW resistance and the extent of the LD around the nearest SNPs. We demonstrated that the VW resistance effect related to the genome region of interest was actually controled by two major QTL and severlal minor loci. The broad-spectrum VW resistance QTL *qVW-D9–1* may be closely linked with the two major QTL. We detected a tandem arrangement of R genes in the redefined confidence interval of *QTL-JESPR0001*. The obtained candidate genes should be helpful in identifying and characterizing defense genes related to VW resistance QTL.

## Additional files

Additional file 1: Table S1. Accession or cultivar, origin, subspecies, type and pedigree of the materials. ^a^ means that the innovation line was created by interspecific hybridization; ^b^ means that the innovation line was created by induced mutagenesis; ^c^ means that the pedigree is unkonwn. (XLS 50 kb)

Additional file 2: Table S2. Physical position of verticillium wilt resistance-related QTL or markers. (XLSX 12 kb)

Additional file 3: Table S3. SNP genotypes of 120 lines. SNPs homologous to the same sequence of the genome region of interest and having the same position when assigning them to the cotton AD genome were noted With yellow coating. (XLSX 1064 kb)

Additional file 4: Table S4. LD between pairs of SNPs. SNPs are indicated by physical position, and the corresponding SNP name is indicated in Additional file 3: Table S3. (XLSX 8341 kb)

Additional file 5: Figure S1. Triangle plots for pairwise LD between SNP markers located in the genome region of interest. (JPEG 48 kb)

Additional file 6: Figure S2. Haplotype blocks of the genome region of interest. (PNG 77 kb)

Additional file 7: Figure S3. Average LnP(D) and *△K* over 5 repeats of STRUCTRUE simulations. (JPEG 23 kb)

Additional file 8: Table S5. Population structure (Q matrix) of the 120 lines. (XLSX 15 kb)

Additional file 9: Table S6. Pairwise kinship estimates of the 120 lines. (XLSX 162 kb)

Additional file 10: Table S7. Statistically significant SNPs associated with VW resistance in the disease nursery environment and in thegreenhouse environment. SNPs detected in both environments were highlighted with yellow. The peak SNPs were highlighted with red. (XLSX 22 kb)

Additional file 11: Table S8. Comparison of VW resistance-associated SNPs with the genome region of interestand detection of functional genes related to VW resistance. (XLSX 18 kb)

## Abbreviations

LD: Linkage disequilibrium
MAF: Minor allelic frequency
MAS: Marker-assisted selection
MLM: Mixed linear model
QTL: Quantitative trait loci
RDI: Relative disease index
VW: Verticillium wilt
