# Supplementary figures and images for: Regional association analysis-based fine mapping of three clustered QTL for verticillium wilt resistance in cotton (G. hirsutum. L)
Source: BMC Genomics. 2017 Aug 25;18:661. doi: 10.1186/s12864-017-4074-y (PMC6389109; doi:10.1186/s12864-017-4074-y)

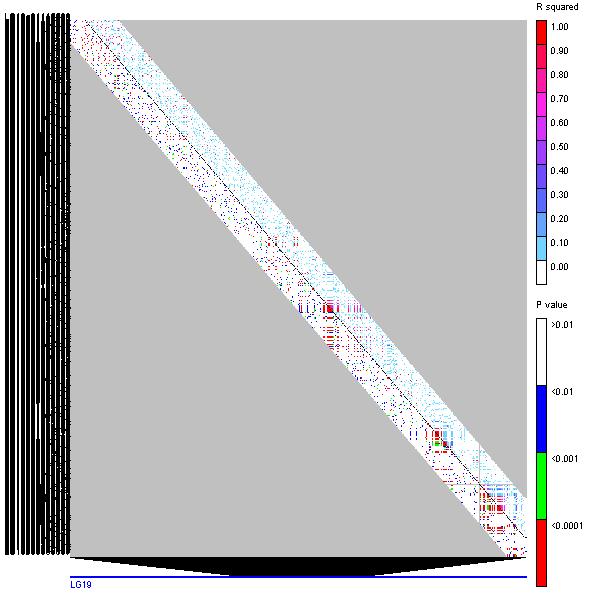

Supplement: Supplementary file 5 — Triangle plots for pairwise LD between SNP markers located in the genome region of interest. (JPEG 48 kb) [file 12864_2017_4074_MOESM5_ESM.jpg]

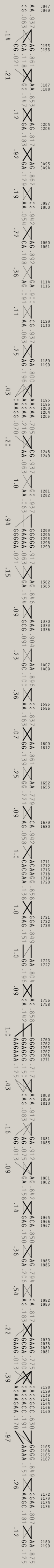

Supplement: Supplementary file 6 — Haplotype blocks of the genome region of interest. (PNG 77 kb) [file 12864_2017_4074_MOESM6_ESM.png]

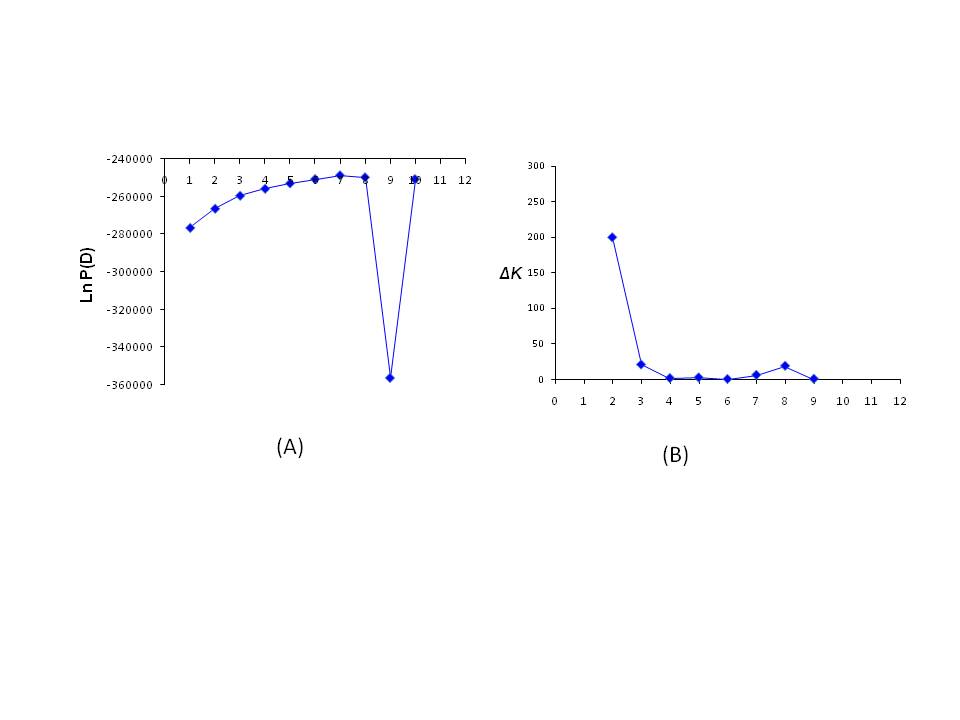

Supplement: Supplementary file 7 — Average LnP(D) and △K over 5 repeats of STRUCTRUE simulations. (JPEG 23 kb) [file 12864_2017_4074_MOESM7_ESM.jpg]
